# Acknowledgement to Reviewers of *Biology* in 2019

**DOI:** 10.3390/biology9020036

**Published:** 2020-02-21

**Authors:** Biology Editorial Office

**Affiliations:** MDPI, St. Alban-Anlage 66, 4052 Basel, Switzerland

The editorial team greatly appreciates the reviewers who have dedicated their considerable time and expertise to the journal’s rigorous editorial process over the past 12 months, regardless of whether the papers are finally published or not. In 2019, a total of 99 papers were published in the journal, with a median time to first decision of 24.5 days and a median time from submission to publication of 56 days. The editors would like to express their sincere gratitude to the following reviewers for their generous contribution in 2019:
Admon, ArieLetsiou, SophiaAlambert, Renata PereiraLewin, AlfredÁlvarez García, GemaLi, MiaoAntony, AntoniouLi, YanAshrafi, AmirmansoorLiu, YangAugenlicht, Leonard H.Llewellyn, Danny J.Baindara, PiyushLuddi, AliceBajwa, AmandeepLuo, MinkuiBaranowska, HannaMacDonald, JessicaBasith, ShaherinMadden, ChristopherBeggel, SebastianMahmud, IqbalBehling, FelixMammadova-Bach, ElminaBenitez, SoniaMao, MengBergstrom, PerMarin Gomez, Oscar H.Bernardo, UmbertoMarino Gammazza, AntonellaBertocchi, MartinaMartínez, GermánBessette, BarbaraMatallanas, BeatrizBhandari, Manohar PrasadMaury, EleonoreBhowmick, SauravMcClary, JillBonanno, GiambattistaMesaros, Clementina A.Bonga, Sjoerd Evert WendelaarMesquita, JoãoBongarzone, ItaliaMijailovich, SrbaBonomini, FrancescaMittag, JensBora, Puran S.Mujtaba, ShirazBorovac, Josip A.Mukherjee, SumitBoucher, JeremieNagahage, IsuraBrady, Matthew J.Narra, Hema PrasadBragado, M. JuliaNaviaux, Robert K.Brauner, ColinNerva, LucaBrew-Appiah, RhodaNesmelov, Yuri E.Bugla-Płoskońska, GabrielaNgamsombat, ChanonBungartz, GerdNikitovic, DraganaBurgstaller, JoergNimmo, HughBush, Stephen J.Norrbom, JessicaCahan, RivkaNotaras, Michael J.Cai, GiampieroNowacka, MalgorzataCambras, TrinitatObermayer, AstridCanfield, ScottOkazawa, AtsushiCarbonell-Bejerano, PabloOster, HenrikCastellano, ImmacolataOstrowski, MartinCharonis, AristidisOtsuka, YoichiroChen, XiaowuPagán, IsraelChen, XiaoyinPage, MelissaCheng, ShixiongPaps, JordiChin, Joong HyounParamythiotis, SpirosChristensen, Emil A.F.Park, Jin-SungCione, ErikaParton, AndrewClausen, Anders R.Passos, MarisaCohen, PaulPatil, AbhinandanColavita, GiampaoloPereiro, PatriciaComi, Giacomo PietroPeruzzotti-Jametti, LucaConn, David BrucePicardo, MauroCorbo, VincenzoPickrell, AliciaCordani, MarcoPillai Raju, RaghavanCorsetti, GiovanniPires, GraçaCsányi, GáborPiwosz, KasiaCsordas, GyorgyPlachetzki, David C.Cui, HuaqingPollard, Thomas DeanDagda, RubenPorter, CraigDague, EtiennePostnova, SvetlanaDangoudoubiyam, SrivenyPramod, SreepriyaDasgupta, SantanuQuinzii, Catarina MariaDe Araujo, ElvinRaetzman, LoriDe FERRON, AntoineRahimi, NaderDe Matteis, RitaRasmussen, LeneDean, AndrewReardon, Catherine A.Delaye, LuisReinke, HansDen Hartigh, Laura J.Ren, LinzhuDesai, AbhishekRodriguez-Vargas, Jose M.Dhar, ArunRosso, GonzaloDinis, Maria Alzira PimentaRout, BhimsenDolgova, OlgaRouyer, FrancoisDöppner, Thorsten R.Rouyer, FrançoisDunlap, JayRoy Chowdhury, SandipanDuston, JimRybka, KrystynaEbeed, HebaSadasivam, MohanrajEggenhofer, ElkeSadoul, BastienEisenback, Jonathan D.Saint-Pol, JulienEl-Esawi, MohamedSaito, KosukeFeregrino-Pérez, Ana A.Saito, MarikoFernández López, IvánSalviati, LeonardoFernández-Mateos, PilarSánchez, Diego HernánFerreira, Paula M.Sandovici, IonelFerrero-Serrano, ÁngelSantos, SusanaFeugang, Jean M.Santulli, GaetanoFischer, FrankSchewe, MatthiasFreire, João A. Schubert, DavidFriedmann Angeli, José PedroSerrão, José EduardoFujii, RitsukoShaw, SubrataFujiwara, SatoruSiebert, Hans-ChristianFuller, ScottSimcox, JudithGagat, MaciejSmith, Doug W.Gallegos, AlbertoSouza, PedroGarstka, Malgorzata AnnaSpanbauer, TrishaGatto, NicoleSprogis, Kate R.Gavrilin, Mikhail A.Srinivasan, BharaniGenders, AmandaStacchiotti, AlessandraGendron, JoshuaStadler, PeterGeoffroy, Cédric G.Stamatos, NicholasGiles, CoreyStanford, KristinGiuliani, MaurizioStiasny, Martina H.Goda, TadahiroStirke, ArunasGrabinska, KarionaSummavielle, TeresaGreischar, Megan A.Sun, YingjieGrignani, GiovanniSurdacki, AndrzejGuan, QingdongSzewczyk-Golec, KarolinaGugger, MurielTan, LiGuo, YanTanase, CorneliuHan, HongyunTanini, MarcoHannah-Shmouni, FadyTatischeff, IrèneHargreaves, IainTauber, EranHatzilazarou, StefanosTeschke, RolfHaufroid, MarieTimoshkin, IgorHenson, JohnTochkov, KirilHiguchi, KeiichiTownsend, KristyHolalu, SrinidhiTriola, GemmaHorai, YoshiroTripathi, Jitendra KumarHoward, Michael J.Troitsky, AlexeyHsu, Yi TingTroyo Diéguez, EnriqueHuang, JiaotiTsikas, DimitrisHughes, CraigTuo, WenbinHuntosova, VeronikaValenzuela, RodrigoIwamoto, HiroyukiVan Der Laarse, Willem J.Iyer, ChitraVan Haren, KeithJ. Vogel, HansVaughan, RogerJain, Shri MohanVayalil, Praveen K.Jefferson, Thomas A.Velez, ZéliaJeffery, GlenVerleih, MariekeJenne, Craig N.Vervaet, BenjaminJoschinski, JensVidal, NievesJoyce, DanielVon Köckritz-Blickwede, MarenKang, Beom SikWan, DongshiKarastergiou, KalypsoWang, FanmiaoKarmali, AminWang, RenKausrud, KyrreWang, XueKavazis, AndreasWard, BessKawai, GotaWarejko, Jillian K.Kennedy, GerardWatterson, StevenKim, DeokWebb, ChailleKim, Yong-IckWedderburn, Scotte D.King, JonathanWeithoff, GuntramKlaus, SusanneWellband, KyleKojima, ShihokoWen, JianjunKokokiris, LambrosWhelan, NathanKolaczkowski, BryanWilliams, David M.Koliesnik, IevgenWilm, MatthiasKozak, Genevieve M.Wilson, RodKozakiewicz, AnnaWright, DavidKriete, AndresWright, DenisKunz, WolframWu, YoujunKusters, IljaXiaoli, AlusLabate, Carlos AlbertoXu, WenliangLakin-Thomas, Patricia L.Yang, Chao-YieLanni, CristinaYasuo, ShinobuLee, Chih-HaoYin, Xiao-MingLee, Joo HyoungYoon, Do-YoungLehnert, KristinaYousefi, ShidaLehnert, MatthewZamora, LeonardoLeidenheimer, NancyZheng, GuopingLeira, ManelZlesak, DavidLemieux, KiTaniŽunar, Bojan

